# Supplementary material for: Redox-coupled proton pumping drives carbon concentration in the photosynthetic complex I
Source: Nat Commun. 2020 Jan 24;11:494. doi: 10.1038/s41467-020-14347-4 (PMC6981117; doi:10.1038/s41467-020-14347-4)
Supplement: Supplementary file 1 — Supplementary Information [file 41467_2020_14347_MOESM1_ESM.pdf]

Supplementary Information

Supplementary Methods

Construction of the *T. elongatus* mutant. The generation of the CupS-TwinStrep-tag (TS) mutant was based on a CupS-StrepII-tag plasmid that was created by PCR amplification of the c*upS* (*tll0220*) gene and the corresponding upstream region using genomic DNA of *Thermosynechococcus elongatus* as template. Thereby at the 3′-end the StrepII-tag coding sequence as well as a SacII restriction site and at the 5'-end a SacI restriction site were fused by primer extensions (primers: P1-*for*-5'-AAAGAGCTCGGCCATAGCCAGATGTC-3'; P2-*rev*-5'-GGGCCGCGGTTATTTTTCGAACTGCGGGTGG CTCCAAGCGCTGACTTTGTAGGGAATGTTG-3'). The product was ligated into a HincII linearized pUC18 plasmid. The *cupS* downstream region was amplified by use of PCR primers fusing a 5' PstI and 3' KpnI site (primers: P3-*for*-5'-GGGCTGCAGGGTATTAGCAGATTT CCC-3'; P4-*rev*-5'-CCCGGTACCCAATCAGGTCGTAGTTGC-3') and afterwards cloned into the plasmid pBluescriptSK(+)Km^R. The kanamycin resistance cassette and the *cupS* downstream region were then ligated via the restriction sites SacII and KpnI into the pUC18 plasmid containing the *cupS* upstream region (see above) resulting in pCupS-StrepII-tag construct. To generate the CupS-TS-tag mutant, *cupS* (*tll0220*) and the corresponding upstream region were amplified by PCR from pCupS-StrepII-tag, and thereby extended by half the sequence of the TS-tag at the 3'-end (primers: P1; P5-*rev*-5'-GCAGAACCACCAGAACCACCGCCGCTGCCGCCGCCTTTTTCGAACTGCGGGTGGC-3'). The product was used as a template for a second PCR and thus extended with the other half of the TS-tag with the addition of the restriction site NotI to the 3'-end (primers: P1; P6-*rev*-5'-CCCGCGGCCGCTTACTTCTCAAAT TGCGGATGAGACCACGCAGAACCACCAGAACCACC-3'). Finally, the StrepII-tag was replaced with the TS-tag via the restriction sites NheI and NotI. The resulting plasmid was transformed into *T. elongatus* according to Ref.[1] Full segregation of the mutant allele was confirmed by PCR (primers: P7-*for*-5'-GGATTAACGGACGCGATTAC-3'; P8-*rev*-5'-AGGCCGATAAGGCAGAACTA-3'). See Supplementary Table 4 for list of primers. The original strain of *Thermosynechococcus elongatus* BP1 originates form Ref. 2.

Culture conditions: *T. elongatus*. The CupS-TS-tag mutant was grown in BG-11 liquid medium[3] with the addition of 80 µg ml^{-1} of kanamycin, at a temperature of 45°C, and under illumination of increasing intensity of 50-200 µmol photons (dependent on cell density). The cells were bubbled with 5% CO_2 until an optical density (OD_{680}) of 1.5 was reached, and then incubated only with compressed air to start the expression of NDH-1MS complex.

Purification of NDH-1MS complex. NDH-1MS was isolated from *T. elongatus* via TwinStrep(TS)/Streptactin affinity chromatography (IBA LifeSciences) with the TwinStrep-tag fused to the C-terminus of CupS (see above). The solubilisation of the thylakoid membranes and the purification of the complex were described earlier in detail.[4,5] Within this study, minor modifications were introduced. In brief, thylakoid membranes were prepared according to Ref.[6] and solubilised with 1% (w/v) glycol-diosgenin (GDN, Anatrace) and buffers for equilibration and elution were prepared with 0.02% (w/v) GDN. Strep-tagged protein complexes were concentrated with a spin concentrator (100 kDa cut off) and stored at -80°C until further analysis. For cryo-EM grid preparation, the sample was polished using size-exclusion chromatography.

Zinc quantification. Zinc quantification was performed by inductively-coupled plasma optical emission spectrometry (ICP-OES) according to Ref. 6. In brief, the dried samples were dissolved in 1 ml 65% (w/w) HNO_3 at RT overnight, incubated at 60°C, 80°C and 100°C for 1 hr each and cooled down to 50°C before addition of 0.2 ml of 30% (v/v) H_2O_2.Then, the sample was heated again to 60°C and 100°C for 30 min each, cooled down to RT and ultrapure water was added to a final volume of 6 ml. The samples were analysed by ICP-OES with an iCAPDuo 6500 instrument (Thermo Fisher Scientific, Dreieich, Germany) after calibration with a multi-element standard. Emission specific for zinc was measured at 2025 nm, 2062 nm and 2138 nm.

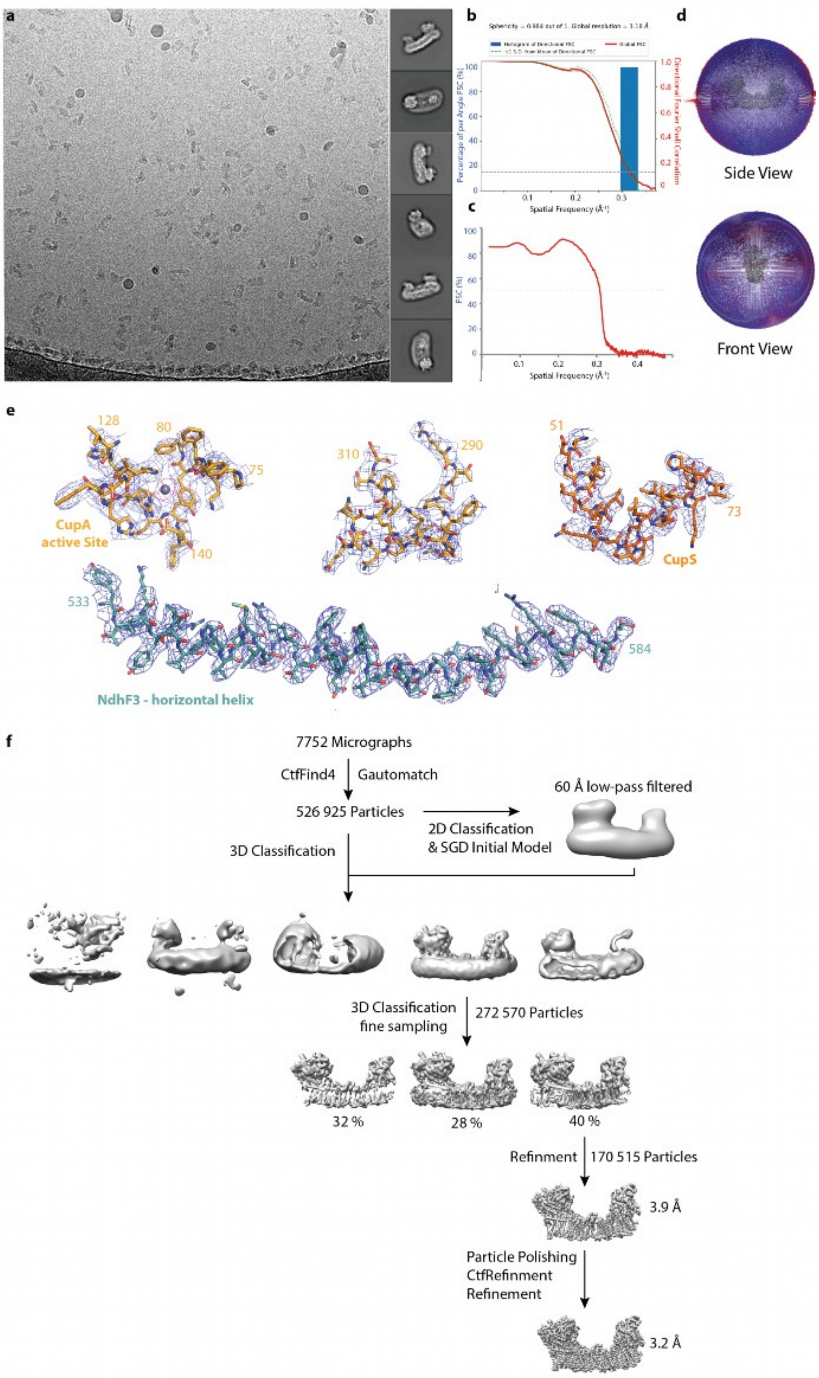

Supplementary Fig. 1 | Cryo-EM data collection, analysis, and classification scheme. a) Representative cryo-EM micrograph collected with an FEI Titan Krios microscope, operated at 300 kV and equipped with a K2 Summit camera. Representative reference-free 2D class averages are shown. **b)** 3DFSC (https://3dfsc.salk.edu) and preferred orientation analysis of the dataset with the red line, representing the estimated global FSC of 3.18 Å ± 1 SD (green dashed lines). A sphericity of 0.984 indicates an isotropic map. **c)** Model vs. map FSC for the final PHENIX real-space refined model. **d)** Angular distribution of the particles used for the final round of refinement. **e)** Representative regions of the photosynthetic complex I (in stick representation, same colours as in Fig. 1) and surrounding electron density maps are shown. Maps are displayed as a mesh using a contour level of up to 2 Å around the atoms. Subunits and residue numbers are specified. **f)** The 526,925 particles selected from the dataset were 3D classified into five classes using as a reference an *ab initio* model generated by the SGD algorithm in RELION. A single class was selected and further sub-classified into three classes. The class consisting of the best aligning particles was subsequently auto-refined in RELION. After Bayesian particle polishing and CTF refinement, the particles were subjected to a final round of 3D refinement in RELION.

a b c

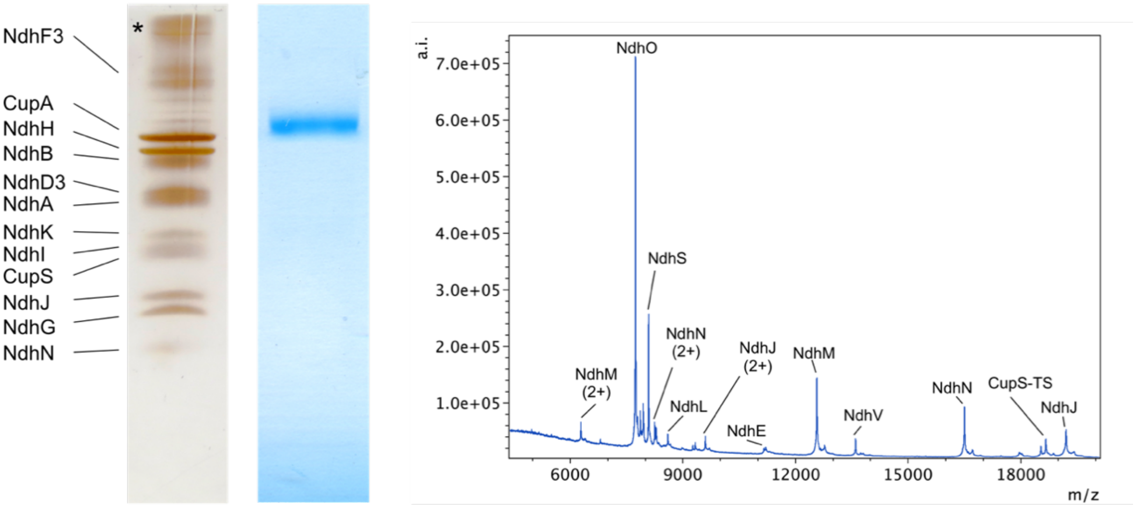

d

	Control (µM)	Sample (µM)
Zn2025	0.03	2.39
Zn2062	0.02	2.39
Zn2138	0.02	2.45
Protein	-	3.33

e

Subunit	Calculated average mass	Measured average mass	Delta mass (Da)	Charge	Modification[a]
NdhJ	19,212	19,213	1	+1	minus M1
CupS	18,668	18,668	0	+1	TwinStrepTag, minus M1
NdhN	16,505	16,505	0	+1	minus M1
NdhV	13,600	13,600	0	+1	M22 (minus M1)
NdhM	12,567	12,566	1	+1	
NdhE	11,161	11,162	1	+1	Formyl-M1
NdhJ	9,606	9,606	0	+2	minus M1
NdhL	8,599	8,601	2	+1	Formyl-M1
NdhN	8,251	8,256	5	+2	
NdhS	8,083	8,084	1	+1	M37 (minus M1)
NdhO	7,736	7,737	1	+1	minus M1
NdhM	6,284	6,285	1	+2	

[a] predicted common modification based on the measured mass and/or corrected first N-terminal amino acid residue

f

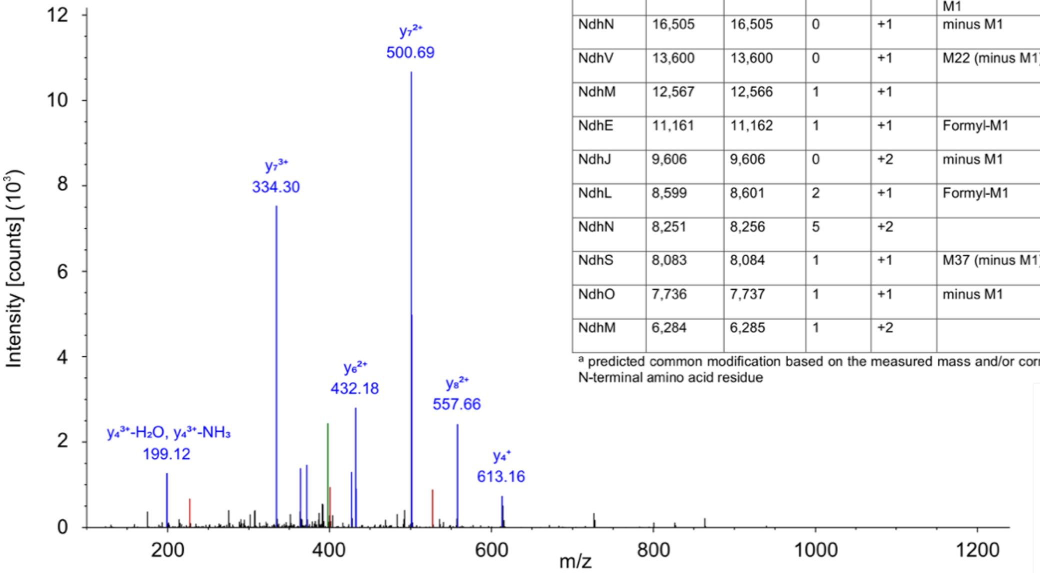

Supplementary Fig. 2 | Subunit composition and mass spectrometry analysis of NDH-1MS from *T. elongatus*. a) The purified NDH-1MS complex was analysed by SDS-PAGE (*probable protein aggregates), **b)** Blue-native PAGE, and **c/e)** MALDI-ToF mass spectrometry as described in Ref.[5] (NdhJ, NdhM and NdnN are present as single and double charged (2+) ions**) d)** Zinc quantification by Inductively-Coupled Plasma Optical Emission Spectrometry (ICP-OES) according to Ref.[6] Zinc was detected at three specific wavelengths (2025 nm, 2062 nm and 2138 nm) and quantified in control (buffer only) and NDH-1MS sample after calibration with a zinc standard. Comparison with the protein concentration indicates a protein-to-zinc ratio of ~0.7. **f)** The CupA protein band (see panel **a**) was digested by trypsin and analysed by tandem mass spectrometry as described in Ref.[5] Citrullination of arginine as well as oxidation of methionine and cysteine were allowed as dynamic modifications and only peptides that were identified with high confidence (false discovery rate >0.01) were considered as positive results. The peptide LLHHLWHDR$_{135}$ was the most abundant peptide (40 peptide spectrum matches vs. 1-23 PSMs) in the analysis and we have no indication for citrullination of Arg135.

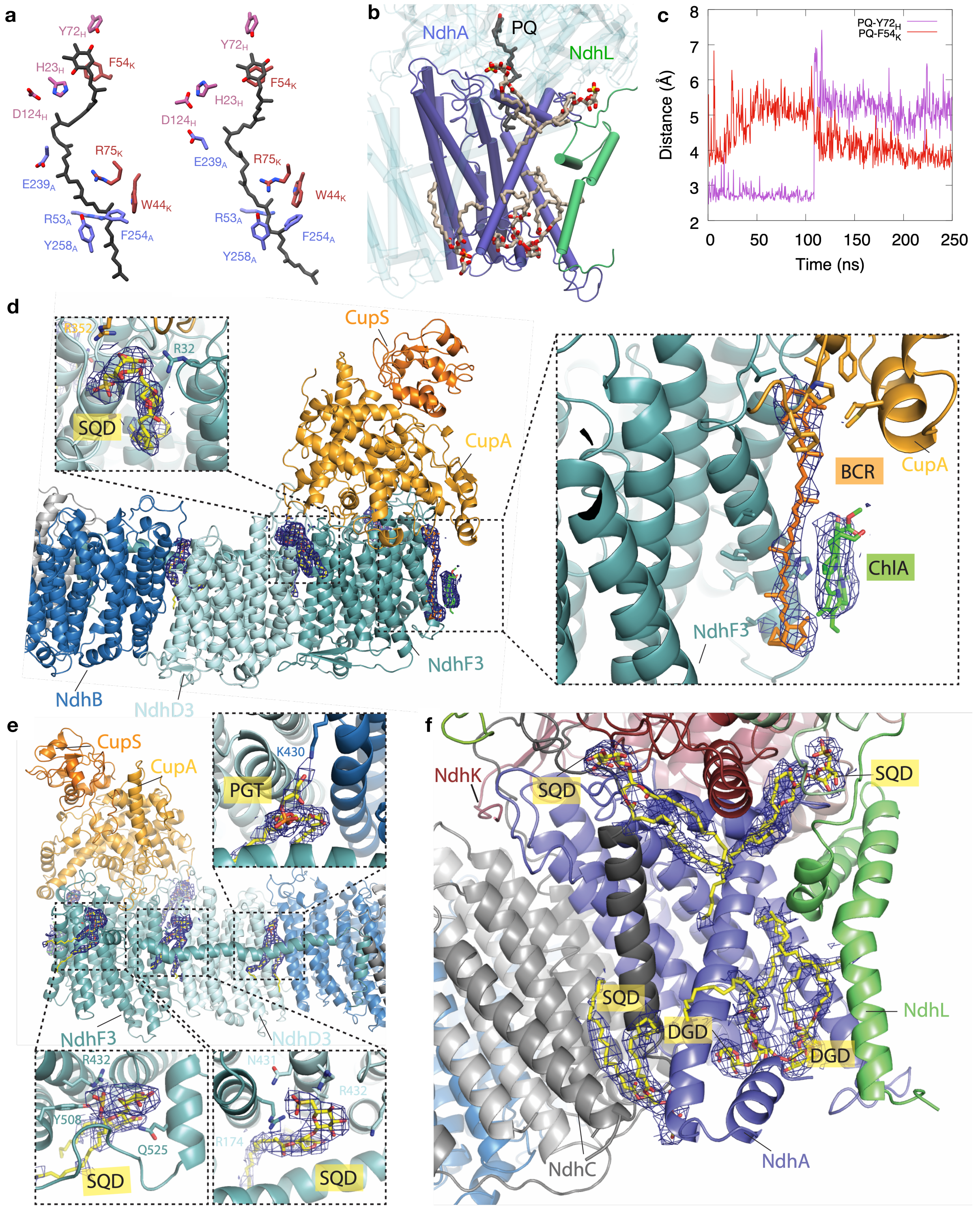

Supplementary Fig. 3 | Non-protein cofactors and lipids in the photosynthetic complex I. a) PQ binding site obtained from MD simulations. PQ in hydrogen-bond interaction with Tyr72 of NdhH subunit (*left*) and in stacking contact with Phe54 of NdhK (*right*). The figures show conserved residues of NdhH, NdhK and NdhA along the PQ-binding cavity. **b)** The PQ-cavity entrance is located in the NdhA subunit at the interface of three α-helices, in the proximity of NdhL (in green), and surrounded by experimentally observed lipid molecules. **c)** Distance of the PQ-headgroup to Tyr72 of NdhH and Phe54 of NdhK during 250 ns MD simulations. **d, e)** Experimentally resolved non-protein cofactors at the NdhF3/CupA interface from **e)** the front and **d)** backview. The binding area for the Chl *a*/β-carotene (ChlA/BCR) motif is magnified. All densities are shown at 3 sigma value and using a contour level of up to 2 Å around the atoms. **f)** Lipid binding next to the PQ entry site. SQD – sulfoquinovosyl diglyceride; DGD - digalactosyl diacylglycerol; PGT – phosphatidylglycerol.

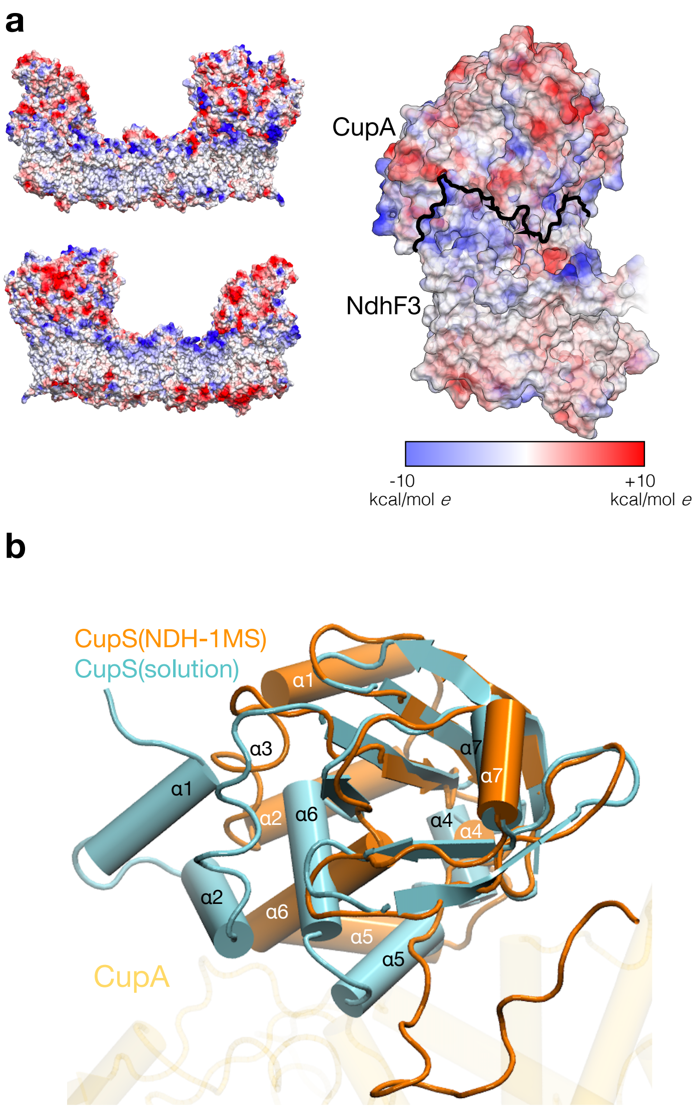

Supplementary Fig. 4 | CupA structure and interactions. a) Electrostatic potential (in kcal/mol *e*) at the surface of the complete NDH-1MS (left) and closeup of the CupA/NdhF3 interface (right). The negative area at the bottom of CupA (red) electrostatically interacts with the positive area (blue) at the top of NdhF3 (CupA-NdhF3 contact shown by a thick black line). **b)** CupS undergoes conformational changes from its solution structure (CupS in cyan, PDB ID: 2MXA[9]) upon binding to CupA (CupS in orange). The figure shows how helices α1, α2, and α3 in the N-terminal region, and α5 and α6 in the middle region, close upon the β-sheet.

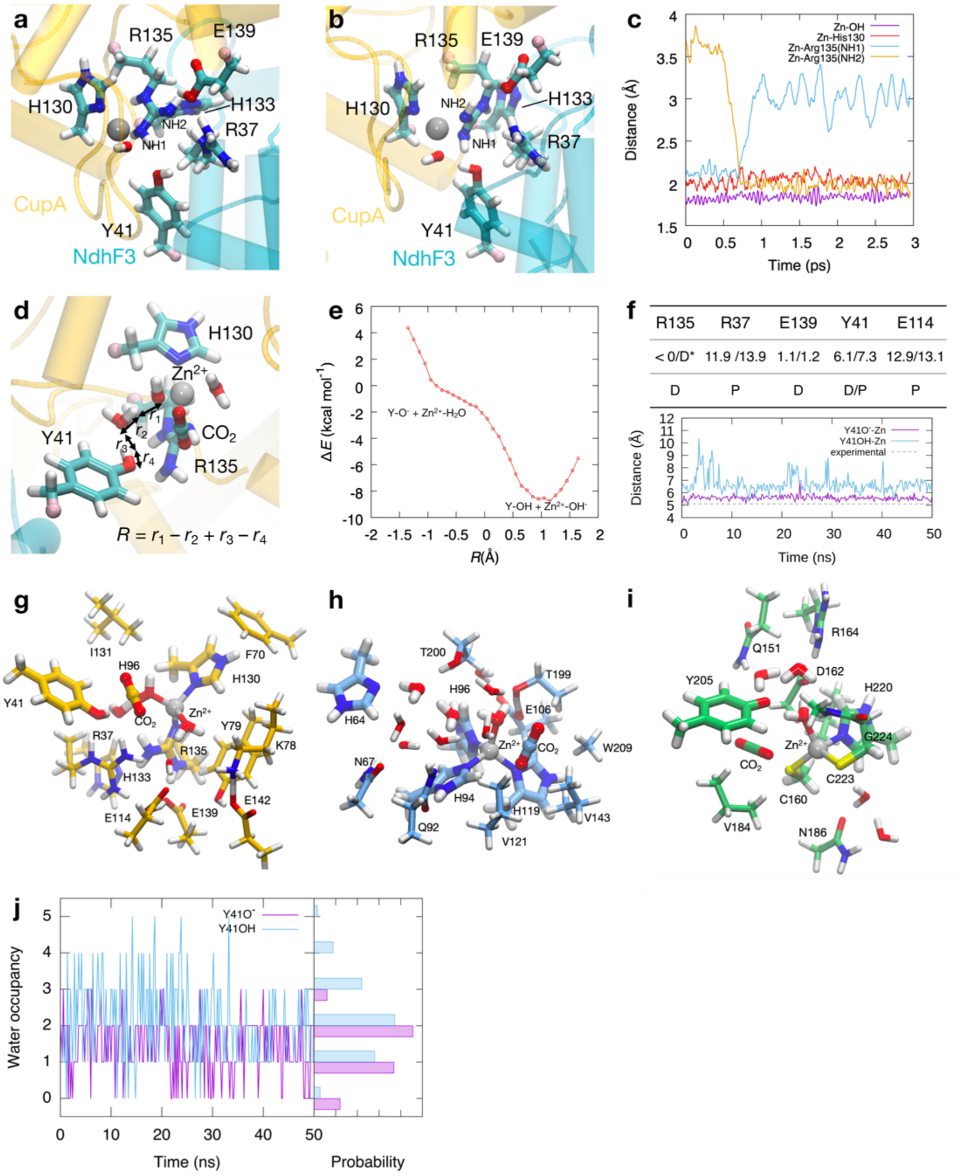

Supplementary Fig. 5 | QM, QM/MM, and PBE calculations on CupA and CA. a-c) QM/MM calculations on a CupA/NdhF3 model. **a)** Arg135 (NH2) deprotonates by proton transfer to Glu139. The NH1 group of Arg135 remains coordinated to Zn^{2+}. **b)** After *ca.* 1 ps QM/MM MD, the NH2 of Arg135 moves closer to Zn^{2+}, further stabilizing the metal centre. **c)** Distance of first coordination sphere ligands to Zn^{2+} during the QM/MM MD simulations. **d, e)** QM/MM calculations of the proton transfer reaction from Zn^{2+}-H_2O to Tyr41, showing **d)** the reaction coordinate, *R,* for the pT process and **e)** the pT energetics. **f)** *Top:* Predicted pK_a values of key residues in CupA and the NdhF3 interface during MDFF relaxation obtained from PBE continuum electrostatic models (see Methods). D - deprotonated residue; P - protonated residue; The second set of values correspond to calculations where R135 has been fixed in its deprotonated state (indicated with asterisk). *Bottom:* Distance of protonated Tyr41 (blue) and deprotonated Tyr41 (purple) to Zn^{2+} during MD simulations, and in the Cryo-EM structure (dashed grey line). **g-i**): DFT models of CO_2 hydration in CupA and CA. Optimised DFT models of **g)** CupA with *N*=185 atoms, **h)** αCA with *N*=155 atoms, and **i)** βCA with *N*=132 atoms. **j)** Water count between Zn^{2+} and Tyr41 during two MD trajectories.

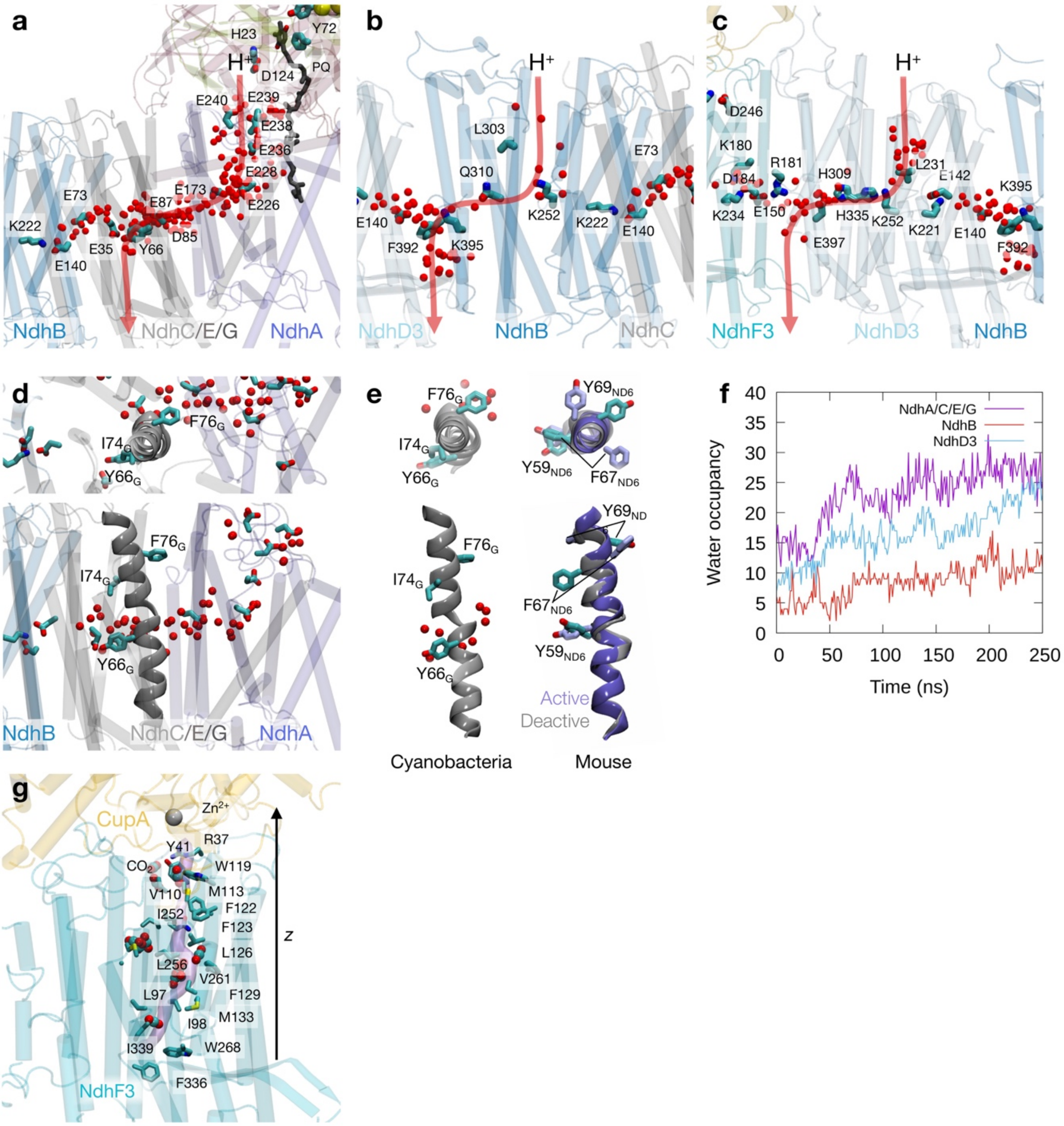

Supplementary Fig. 6 | Proton and CO_2 channels formed during MD simulation. The figure shows water molecules (red sphere) averaged over 250 ns MD simulations in **a)** NdhC/E/G, **b)** NdhB, and **c)** NdhD3. **d)** Proton channel in NdhA/E/G and comparison to TM3 rotation in mouse complex I.[10] **e)** TM3 of NdhG in NDH-1MS (grey, *left*). TM3 helix of ND6 of complex I from *Mus musculus* (*right*) in the deactive (grey) and active (purple) states. **f)** Hydration dynamics in the putative proton channels in subunits NdhB, NdhD3, and NdhA/C/E/G during MD simulations. **g)** The putative CO_2 gas channel (purple) is surrounded by hydrophobic and bulky residues, and connects to the Zn^{2+}-binding site by conserved Tyr41, Arg37 and Glu114 of NdhF3 subunit.

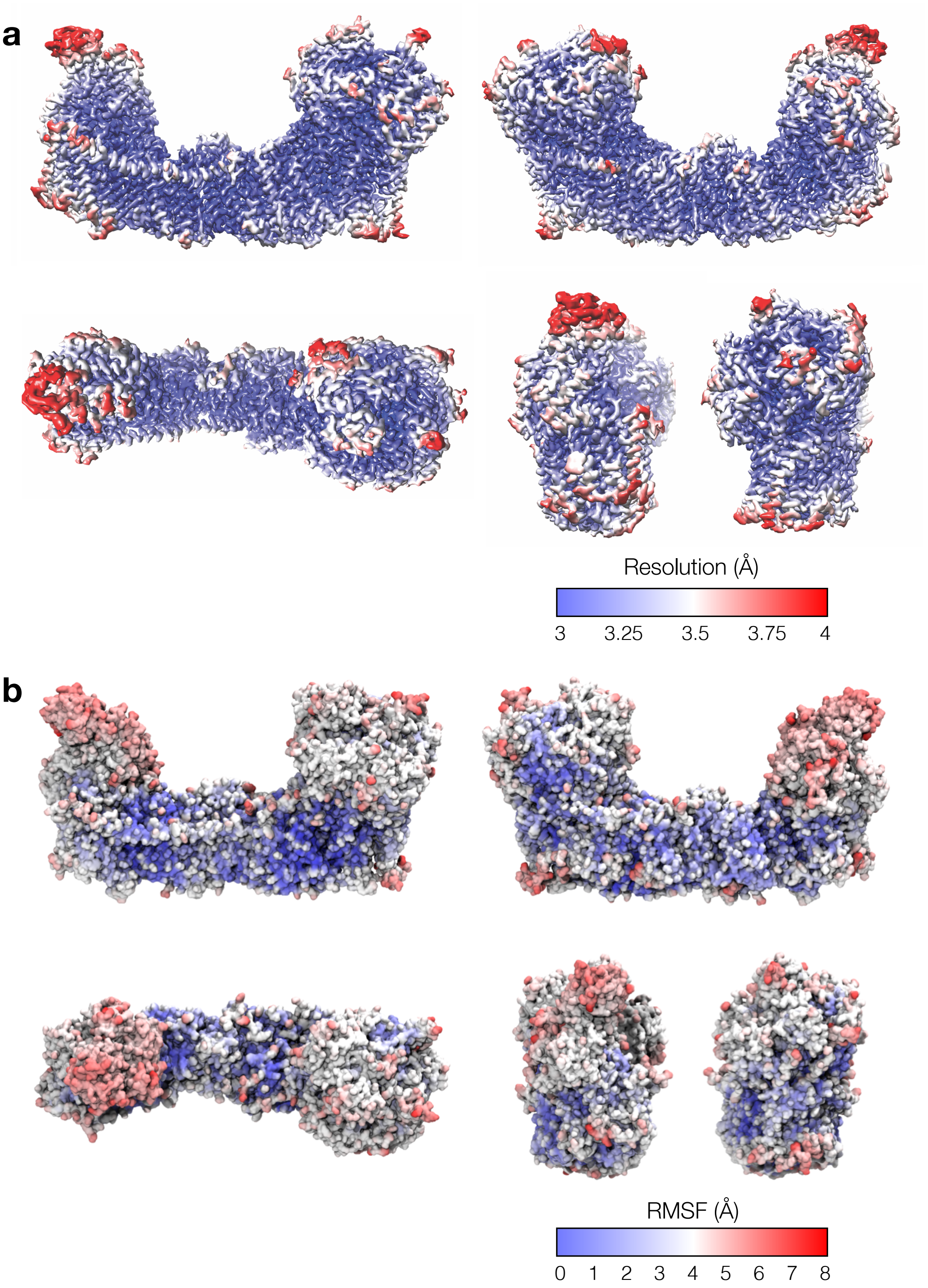

Supplementary Fig. 7 | Dynamics of NDH-1MS inferred from the local resolution of the cryo-EM map and from MD simulations. a) The resolution was estimated using the local resolution function in RELION with default parameters and plotted using UCSF Chimera. Units are in (Å). **b**) Root-mean-square-fluctuations (RMSF, in Å) obtained from 250 ns MD simulations of NDH-1MS.

Supplementary Table 1 | Cryo-EM data collection, refinement and validation statistics.

	NDH-1MS (EMDB-10513) (PDB ID: 6TJV)
**Data collection and processing**	
Magnification	105.000 x
Voltage (kV)	300
Electron exposure (e–/Å^2)	40.2
Defocus range (μm)	0.5-3.5
Pixel size (Å)	1.35
Symmetry imposed	C1
Initial particle images (no.)	526.925
Final particle images (no.)	170.151
Map resolution (Å)	3.20
FSC threshold	0.143
Map resolution range (Å)	2.98-7.26
**Refinement**	
Initial model used (PDB code)	6HUM, *de-novo*
Model resolution (Å)	3.23
FSC threshold	0.5
Model resolution range (Å)	3.23
Map sharpening *B* factor (Å^2)	-89.89
Model composition	
Non-hydrogen atoms	68541
Protein residues	4275
Ligands	15
B factors (Å^2)	
Protein	60.9
Ligand	64.8
R.m.s. deviations	
Bond lengths (Å)	0.007
Bond angles (°)	1.109
Validation	
MolProbity score	2.2
Clashscore	11
Poor rotamers (%)	1.29
Ramachandran plot	
Favored (%)	88.00
Allowed (%)	11.48
Disallowed (%)	0.52

Supplementary Table 2 | Tandem-LC-MS analysis of NDH-1MS. For BN-PAGE, see Supplementary Fig. 2b.

Subunit[a]	ORF	Mass (kDa)[b]	TMH[c]	XC[d]	Coverage[e]
NdhA	tlr0667	41.3	8	49.66	18.47
NdhB	tll0045	55.1	14	59.88	7.57
NdhC	tlr1429	15.0	3	4.67	15.91
NdhD	tlr0905	53.8	14	20.74	5.59
NdhE	tlr0670	11.1	3	20.05	14.85
NdhF	tlr0904	66.2	16	69.12	7.86
NdhG	tlr0669	21.6	5	5.35	16.50
NdhH	tlr1288	45.2	-	302.11	49.24
NdhI	tlr0668	22.4	-	151.24	57.14
NdhJ	tlr1430	19.3	-	142.17	59.52
NdhK	tlr0705	25.7	-	129.30	29.11
NdhL	tsr0706	8.6	2	5.42	11.84
NdhM	tll0447	12.6	-	133.40	49.55
NdhN	tlr1130	16.6	-	59.28	55.33
NdhO	tsl0017	7.9	-	27.22	57.14
NdhS	tlr0636	8.2	-	18.51	62.16
NdhV	tlr0472	13.7	-	10.34	19.20
CupA	tlr0906	50.9	-	268.37	49.24
CupS-TS	tll0220	18.8	-	109.15	55.31

[a] results were filtered for known NDH-1MS subunits
[b] calculated molecular weight
[c] number of transmembrane helices
[d] sequest protein score
[e] sequence coverage (percent)

```
T. elongatus (BP-1)/1-437                  1 MVQAMERPSSAKLPPLDHPLADIIYRLEAGGALIPDTPVNLMKIIGMYKAYSIPMDFYWRDLLYLGERVFINPFPFFKYFPTKEYFEL  88
Synechocystis sp. (PCC 6830)/1-431         1 ---MTTLTPKATLPPSTHPFADVIHRLEAGGSMLPDTPENLMQIIGIYKAYAVPMDFYWRDLLYIAERVFLNPLRFFKYFLPQEYLDL  85
Synechococcus sp. (PCC 6301)/1-430         1 ---MTVTTRTTPLPPSQHRFAEVIHRLEAGGSMLPDTPDNLKQIIGIYKAYAVPMDFYWRDLLYIAEQVFLNPIPAFKYFISQEYLDR  85
M. aeruginosa (TAIHU98)/1-431              1 ---MTGTLIKAKIPPSTHEFADIIHRLEAGGSMLPDTPENLMQIIGIYKAYAVPMDFYWRDLLYIAERVFLDPLPFFKYFLPQEYLDL  85
Thermosynechococcus sp.(NK55a)/1-437       1 MVQAMERPSSAKLPPLDHPLADIIYRLEAGGALIPDTPVNLMKIIGMYKAYSIPMDFYWRDLLYLGERVFINPFPFFKYFPTKEYFEL  88
S. elongatus (PCC 7942)/1-430              1 ---MTVTTRTTPLPPSQHRFAEVIHRLEAGGSMLPDTPDNLKQIIGIYKAYAVPMDFYWRDLLYIAEQVFLNPIPAFKYFISQEYLDR  85
S. cyanosphaera (PCC 7437)/1-434           1 MVQTPIKQPTAKLPPSTHQFADVIHRLEAGGSMLPDTPENLMQIIGIYKAYAVPMDFYWRDLLYIAERVFLEPLPFFKYFLPKEYLDL  88
Cyanothece sp. (ATCC 51142)/1-433          1 MVQTPIK-PTGIIPPSNHQFAEVIYRLEAGGSMLPDTPENLMQIIGIYKAYAVPMDFYWRDLLYIAERVFLDPLPFFKYFLPKEYLDL  87
Xenococcus sp. (PCC 7305)/1-433            1 MVQT-SPKSTSKIPPSTHEFADVIHRLEAGGSMLPDTPENLMQIIGIYKAYAVPMDFYWRDLLYIAERVFLNPLPFFKYFLPQKYLDL  87
Nostoc_sp._(PCC_7524)/1-440                1 MVQ-TPDKPVTKIPPSQHEFADIIYRLEAGGSMLPDTPENLMQIIGIYKAYAVPMDFYWRDLLYIAEQVFLNPFSFFKYFISKEYLER  87

T. elongatus (BP-1)/1-437                 89 PNHYAGDTADLRIWRGPAHAHPELMEFIEKGET-GKMPRLLHHLWHDRINMEFSEDLARAMMWHRM----GGQLDIYLDSEEYKAAAD 171
Synechocystis sp. (PCC 6830)/1-431        86 ANHYAGETADLRIWRGEASAHPELLEFMEKGNT-TKMPKLFHHLWHDRINMEFAEACMQAMLWHGRDM-GMGKFDTYLDSDEYKANAD 171
Synechococcus sp. (PCC 6301)/1-430        86 PNSYAGDNADLRIWRGPATAHPELLEFMQKGELKRKLPKWLHHLWHDRVNMEFAEACMDAMLWHQGM---GGRFNDYLESEEYRQNAD 170
M. aeruginosa (TAIHU98)/1-431             86 QNHYAGDNADLRIWRGTGSAHPELLEFMDKGKT-RKMPRLIHHLWHDRINMEFAEACMQAMLWHGRDM-GWGLFDAYLDSEEYRQNAD 171
Thermosynechococcus sp.(NK55a)/1-437      89 PNHYAGDTADLRIWRGPAHAHPELMEFIEKGET-GKMPRLLHHLWHDRINMEFSEDLARAMMWHRM----GGQLDIYLDSEEYKAAAD 171
S. elongatus (PCC 7942)/1-430             86 PNSYAGDNADLRIWRGPATAHPELLEFMQKGELKRKLPKWLHHLWHDRVNMEFAEACMDAMLWHQGM---GGRFNDYLESEEYRQNAD 170
S. cyanosphaera (PCC 7437)/1-434          89 HNHYAGDDADLRIWRGEATAHPELLEFMEKGKT-FKMPKLFHHLWHDRINMEFAEACMRAMLWHGRDM-GWGKFDAYLDTEEYKANAD 174
Cyanothece sp. (ATCC 51142)/1-433         88 HNHYAGDDADLRIWRGEATAHPELLEFMEKGKT-GKLPKLFHHLWHDRINMEFAEACMRAMLWHGRDM-GWGKFDAYLDSEEYKANAD 173
Xenococcus sp. (PCC 7305)/1-433           88 PNHYAGENADLQVWRGRASAHPELLEFMEKGTT-SKMPKLFHHLWHDRINMEFAEACMRAMLWHGRDM-GWGKFDAYLDTEEYKANAD 173
Nostoc_sp._(PCC_7524)/1-440               88 HNHYAGDDADLRIWRGEATAHPELLAFMEKGET-FKIPKLLHHLLHDRINMEFAEACMRAMLWHRHMYAPVNQFDAYLDSEEYKANAD 174

T. elongatus (BP-1)/1-437                172 KAIRAYFKRNPLMLGLYKLFPDLFLEQARQATYMNVLGLFWEVMAPVFFEISDRYDEGSITSVKDAMNFLVNGIFAIAGRPIYHHVYI 259
Synechocystis sp. (PCC 6830)/1-431       172 KAIRAYFKGNPPMLALYKLFPDLFLEQVRELSYYSNLGLFWEVMAPVFFEMSDIYDEGGFKGVPDAMNFLVNGIFAIAGRPIYHHVYI 259
Synechococcus sp. (PCC 6301)/1-430       171 RAIRAYFQGNPLMLGLYKLFPEMFLEQVRMLSYTANLGLFWEVMAPVFFEMSDIYDEGGFKGVPDAMNFLVNGIFAIAGRPIYHHVFI 258
M. aeruginosa (TAIHU98)/1-431            172 RAIKAYFKKNPVMMKLYDLFPDMFLEQVRMMSYYSNLGLFWEVMAPVFFEMSDIYDEGGFKGVPDAMNFLVNGIFAIAGRPIYHHVYI 259
Thermosynechococcus sp.(NK55a)/1-437     172 KAIRAYFKRNPLMLGLYKLFPDLFLEQARQATYMNVLGLFWEVMAPVFFEISDRYDEGSITSVKDAMNFLVNGIFAAAGRPIYHHVYI 259
S. elongatus (PCC 7942)/1-430            171 RAIRAYFQGNPLMLGLYKLFPEMFLEQVRMLSYTANLGLFWEVMAPVFFEMSDIYDEGGFKGVPDAMNFLVNGIFAIAGRPIYHHVFI 258
S. cyanosphaera (PCC 7437)/1-434         175 RAIKAYFKYNPAMLGLYKLFPDMFLEQVRQLSYYSNLGLFWEVMAPVFFEMSDIYDEGGFKGVPDAMNFLVNGIFAIAGRPIYHHVYI 262
Cyanothece sp. (ATCC 51142)/1-433        174 KAIKAYFKHNPAMLGLYKLFPDMFLEQVRQLSYYSNLGLFWEVMAPVFFEMSDIYDEGGFKGVPDAMNFLVNGIFAIAGRPIYHHAYI 261
Xenococcus sp. (PCC 7305)/1-433          174 RAIKAYFKGNPLMLGLYKLFPDMFLEQVRQLSYYSNLGLFWEVMAPVFFEMSDIYDEGGFKGVPDAMDFLVNGIFAVAGRPIYHHVYI 261
Nostoc_sp._(PCC_7524)/1-440              175 KAIKAYFQGNPVMLGMYKLFPDMFLEQCRQMSYYANLGLFWEVMAPVFFEMSDIYDEGGFKGVPDAMNFLVNGIFAVAGRPIYHHVYI 262

T. elongatus (BP-1)/1-437                260 DDEVHVLVPKEKGFMWLYEAAFPYVEAVFYRTSPFRGTKSYNAQANQVPTDQVDFHYGILFADKFPVGTAGIPPTLLHQDMYHFLPQY 347
Synechocystis sp. (PCC 6830)/1-431       260 DGECLEIIPKSKGFTWLYEAALPYVEAVFYRTAPFRGTKSYNAQAKQVPEEQKDFHYGILYADVFPVGTAGIPPTLLMDDMLHFLPPY 347
Synechococcus sp. (PCC 6301)/1-430       259 NGECFEIIPKSAGFTWLYEAALPYVEAVFYRTAPFRGTKSYNAQAQQVPDQQADFHYGILYADVFPVGSAGIPPTLLMQDMLHFLPPY 346
M. aeruginosa (TAIHU98)/1-431            260 DGQCYEIIPKSKAFTWLYEAALPYVEAVFYRTAPFRGTKSYNAQAKQVPDEQKDFHYGILYADVFPVGTAGIPPTLLMDDMYHFLPQY 347
Thermosynechococcus sp.(NK55a)/1-437     260 DDEVHVLVPKEKGFMWLYEAAFPYVEAVFYRTSPFRGTKSYNAQANQVPTDQVDFHYGILFADKFPVGTAGIPPTLLHQDMYHFLPQY 347
S. elongatus (PCC 7942)/1-430            259 NGECFEIIPKSAGFTWLYEAALPYVEAVFYRTAPFRGTKSYNAQAQQVPDQQADFHYGILYADVFPVGSAGIPPTLLMQDMLHFLPPY 346
S. cyanosphaera (PCC 7437)/1-434         263 RGECYEIIPKSKGFTWLYEAALPYVEAVFYRTAPFRGTKSYNAQAKQVPDEQKDFHYGILYADVFPVGTAGIPPTLLMDDMYHFLPDY 350
Cyanothece sp. (ATCC 51142)/1-433        262 RGECYEIIPKSKGFTWLYEAALPYVEAVFYRTAPFRGTKSYNAQAKQVPDEQKDFHYGILYADVFPVGTAGIPPTLLMDDMYHFLPDY 349
Xenococcus sp. (PCC 7305)/1-433          262 DDECYEIIPKSQGFTWLYEAALPYVEAVFYRTAPFRGTKSYNAQAEQVPARQKDFHYGILYADVFPVGTAGIPPTLLMDDMFHFLPDY 349
Nostoc_sp._(PCC_7524)/1-440              263 RGECYEIIPKSKGFTWLYEAALPYVEAVFYRTAPFRGTKSYNAQAGQVPDDQKDFHYGILYADVFPVGTAGIPPTLLMQDMLHFLPQY 350

T. elongatus (BP-1)/1-437                348 LKDYFHQHCRGEDDILVQLGIAFQHAMYTVTSAVLQATRAAFYYPLDDPNPEHLMANRRFFVAQMDRFLRPQYGIAEACKIRNVQDPN 435
Synechocystis sp. (PCC 6830)/1-431       348 LIEYYQKHCRGEDDMLVQLGITFQRSMYNVTSAVIQALRTALLYPLDDPNPRHLAKNRQFFEAQIDRFLRPE------ARLKDIQSDS 429
Synechococcus sp. (PCC 6301)/1-430       347 LLEEYRQHCRGEDDILIQLGVTFQRSMYNVTSAVIQALRAALLYPLDDTDPVHLQANRAFFEAQMDRFLRPE------ARLPDIQSQD 428
M. aeruginosa (TAIHU98)/1-431            348 LIDYYQQQCRGQDDILIQLGISFQRSMYNVTSAVIQALRTALLYPLDDPNPKHLLKNRQFFEAQMDRFKRPE------ARLRDIQSPN 429
Thermosynechococcus sp.(NK55a)/1-437     348 LRDYFHQHCRGEDDILVQLGIAFQHAMYTVTSAVLQATRAAFYYPLDDPNPEHLMANRRFFVAQMDRFLRPQYGIAEACKIRNVQDPN 435
S. elongatus (PCC 7942)/1-430            347 LLEEYRQHCRGEDDMLIQLGVTFQRSMYNVTSAVIQALRAALLYPLDDTDPVHLQANRAFFEAQMDRFLRPE------ARLPDIQSQD 428
S. cyanosphaera (PCC 7437)/1-434         351 LQKYYQEHCRGEEDVLIQLGITFQRSMYNVTSAVIQALRQALLYPLDDQNPKHLMKNRAFFEAQMDRFLRPE------ARLKDIQSQD 432
Cyanothece sp. (ATCC 51142)/1-433        350 LIEYYQKHCRGEQDILIQLGITFQRSMYNVTSAVIQALRQALLYPLDDPNPQHLLKNRQFFEQQMDRFVRPE------ARLRDIQNQE 431
Xenococcus sp. (PCC 7305)/1-433          350 LQEYYEQNCRGEDDVLIQLGITFQRSMYNVTSAVIQALRQALLYPLDDENPKHLMKNRAFFESQVDRFLRPE------ARLRDIQSQD 431
Nostoc_sp._(PCC_7524)/1-440              351 LVDYYKQYCRGEEDTLIQLGISFQRSMYNVTSAVIQALRTALLYPLDDPNPKHLQANREFFEAQLSRFTRSEYGMRNAARLRDVQSQD 438

T. elongatus (BP-1)/1-437                436 YL                                                                                       437
Synechocystis sp. (PCC 6830)/1-431       430 YR                                                                                       431
Synechococcus sp. (PCC 6301)/1-430       429 YR                                                                                       430
M. aeruginosa (TAIHU98)/1-431            430 YR                                                                                       431
Thermosynechococcus sp.(NK55a)/1-437     436 YL                                                                                       437
S. elongatus (PCC 7942)/1-430            429 YR                                                                                       430
S. cyanosphaera (PCC 7437)/1-434         433 YR                                                                                       434
Cyanothece sp. (ATCC 51142)/1-433        432 YR                                                                                       433
Xenococcus sp. (PCC 7305)/1-433          432 YR                                                                                       433
Nostoc_sp._(PCC_7524)/1-440              439 YR                                                                                       440
```

Supplementary Table 3 | Multiple sequence alignment (MSA). of the CupA (top) and NdhF (bottom). Colouring conservation threshold: 50%. Colouring method: clustal. Colouring conservation threshold: 90%. The MSAs were performed with ClustalW.[8]

```
NdhF3 T. elongatus/1-611            1 MLQSFADTVWLIPFYSLAGMVLS----LIWSPGITRKTGPRPAGY--LNILLTFFSFVHALLATVAIANQ--PPQYLHWTWLDVAGLHLD  82
NdhF4 T. elongatus/1-609            1 MPNSLLETSWWVPCYGLVGAALT----LPWATGYVRRTGPRPAAY--FNLLMTVVAFGHGFFLLQQTRSG--ATATIVWHWLQAPGLDLS  82
NdhF1 T. elongatus/1-656            1 -MEPLYQYAWLIPVLPLLGALIVGFGLIAFSETTSKLRRPSAIFIM----ALMAIAMGHSLTLFWSQVQGHLPYT-QMIEWAAAGNLHIA  84
NdhF3 Synechocystis sp./1-615       1 MLESLSRIIWLVPCYALLGALLA----VPWSPGLTRQTGPRPAGY--ISTLMTFVAFLHSLLVLIHIWQQ--PAIDLSFPWLHAADLEIN  82
NdhF4 Synechocystis sp./1-634       1 MSDFLLQSSWFIPFYGLIGSILS----LPWSFRLIKQTGPRPAAY--FNVFMTLVSAIHGMVALSAIWQT--PSEQIVFHWLQVADLDLT  82
NdhF1 Synechocystis sp./1-681       1 -MELLYQLAWLIPVLPLFGATVVGIGLISFNQATNKLRQINAVFII----SCLGAALVMSGALLWDQIQGHASYA-QMIEWASAGSFHLE  84
Nqo12 T. thermophilus/1-606        1 ------MALLGTILLPLLGFALLGLFGKRMREPLPGVLASGLVL----ASFLLGAGLL---------LSGGARFQA---EWL----PGIP  64
NuoL E. coli/1-613                1 -----MNMLALTIILPLIGFVLLAFSRGRWSENVSAIVGVGSVGLAALVTAFIGVDFF---------ANGEQTYSQPLWTWMSVGDFNIG  76
ND5_M._musculus/1-607             1 -MNIFTTSILLI-FILLLSPIL-----ISMSNLIKHINFP------LYTTTSIKFSFIISLLPLLMFFHNNMEYMITTWHWVTMNSMELK  77

NdhF3 T. elongatus/1-611           83 IPVEISILTTTALMLITALNLMAQVFAVGYMEMDWGWARFFALLALFEGGMGALVLLDSLFFNYVVLEILTLATYLLIGLWFNQPLVVTG 172
NdhF4 T. elongatus/1-609           83 FSLLINSVTTGAMELVTGLSILAQIFALGYLEKDWGMARFFALMGFFEAALSGIAISDSLLLSYGLLEMLTLSTYLLIGFWYAQPLVVKA 172
NdhF1 T. elongatus/1-656           85 MGYVIDPLAALMLVIVTTVAFLVMLYSDGYMAHDPGYVRFFAYLSLFGSSMLGLVVSPNLVQVYIFWELVGMCSYLLIGFWYDRKSAAEA 174
NdhF3 Synechocystis sp./1-615      83 FDLKISTVNIAALVLITGLNLGAQIYAIGYLERDWGWARFFSLMALFEAGLCTLVLCNSLFFSYVVLEILTLGTYLLIGYWFNQSLVVTG 172
NdhF4 Synechocystis sp./1-634      83 LAVEISPVSLGALSVVTGISFLVQIFGLGYMEKDWSLARFYGLLGFFEAALGGIALSDSLFLSYGLLEMLTLSTYLLVGFWYAQPLVVTA 172
NdhF1 Synechocystis sp./1-681      85 MGYVIDHLSALMLVIVTSVALLVMIYTDGYMAHDPGYVRFYAYLSLFASSMLGLVISPNLVQVYIFWELVGMCSYLLIGFWYDRKAADA 174
Nqo12 T. thermophilus/1-606       65 FSLLLDNLSGFMLLIVTGVGFLIHVYAIGYMGGDPGYSRFFAYFNLFIAMMLTLVLADSYPVMFIGWEGVGLASFLLIGFWYKNPQYADS 154
NuoL E. coli/1-613               77 FNLVLDGLSLTMLSVVTGVGFLIHMYASWYMRGEEGYSRFFAYTNLFIASMVVLVLADNLLLMYLGWEGVGLCSYLLIGFYYTDPKNGAA 166
ND5_M._musculus/1-607            78 MSFKTDFFSILFTSVALFVTWSIMQFSSWYMHSDPNINRFIKYLTLFLITMLILTSANNMFQLFIGWEGVGIMSFLLIGWWYGRTDANTA 167

NdhF3 T. elongatus/1-611          173 ARDAFLTKRVGDLVLLMGVLAIYPLAGSWNYDDLAAWAATAQV----NSTLITLICLALIAGPMGKCAQFPLHLWLDEAMEGPIPASIL- 257
NdhF4 T. elongatus/1-609          173 ARDAFLTKRVGDILLLMGVVALGSLAGSYDFPHLYEWAEQANL----PDGWGFLLGLALIAGPTGKCAQVPLHLWLDEAMEGPNPASIM- 257
NdhF1 T. elongatus/1-656          175 AQKAFVTNRVGDFGLLLGMVGLFWATGTFDFAGMGDRLTELVNTGLLSPSLAAILAILVFLGPVAKSAQFPLHVWLPDAMEGPTPISALI 264
NdhF3 Synechocystis sp./1-615     173 ARDAFLTKRVGDLILLMGVVALLPLAGSWNYDDLAQWAASADL----NPTAATLLCLALIAGPLAKCAQFPLHLWLDEAMEGPIPATIL- 257
NdhF4 Synechocystis sp./1-634     173 ARDAFLTKRVGDIILLMGVVALSSYGQGLTFSQLDNWASTVPV----TGITATLLGLSLIAGPTGKCAQFPLNLWLDEAMEGPNPAGIM- 257
NdhF1 Synechocystis sp./1-681     175 CQKAFVTNRVGDFGLLLGILGLYWATGSFDFGTIGERLEGLVSSGVLSGAIAAILAILVFLGPVAKSAQFPLHVWLPDAMEGPTPISALI 264
Nqo12 T. thermophilus/1-606      155 ARKAFIVNRIGDLGFMLGMAILWALYGTLSISELKEAMEGPLK-N---PDLLALAGLLLFLGAVGKSAQIPLMVWLPDAMAGPTPVSALI 240
NuoL E. coli/1-613              167 AMKAFVVTRVGDVFLAFALFILYNELGTLNFREMVELAPAHFADG---NNMLMWATLMLLGGAVGKSAQLPLQTWLADAMAGPTPVSALI 253
ND5_M._musculus/1-607           168 ALQAILYNRIGDIGFILAMVWFSLNMNSWELQQ---IMFSNNN-----DNL--IPLMGLLIAATGKSAQFGLHPWLPSAMEGPTPVSALL 247

NdhF3 T. elongatus/1-611          258 RNAVVVATGAWVLVKLTPVLSLSPVALTALLVIGSVTALGGTLIAIAQVDIKRALSYLVSAYMGWVFIAVGLKEPGLAFVFILTYSLAMA 347
NdhF4 T. elongatus/1-609          258 RNSVVVAAGAYILIKLQPILVACPGANIALIAIGTVTAISESLVSIAQIDIKRALSHSTSVYLGLVFIGVGTNWTDFALFVLLTHAIAKA 347
NdhF1 T. elongatus/1-656          265 HAATMVAAGVFLIARMFPVFEQLPQVMTTIAWTGAFTAFMGATIAITQNDIKKSLAYSTISQLGYMVMGMGVGAYSAGLFHLMTHAYFKA 354
NdhF3 Synechocystis sp./1-615     258 RNTLVVATGAWVLVKVQPILALSPVALTVMIAIGSVTAIGASLIAIAQIDIKRFLSYVVSAYMGLVFIAVGTGQGETALQLIFTYTFAMA 347
NdhF4 Synechocystis sp./1-634     258 RNSVVVSAGAYVLIKLQPVFTLSPIASKTLIVLGTLTVVMTSLIAIAQIDIKRTLSHSTSVYLGLVFIAVGLGQVDIAFLLLFAHAIAKA 347
NdhF1 Synechocystis sp./1-681     265 HAATMVAAGVFLVARMYPVFEPIPVVMNTIAFTGCFTAFLGATIALTQNDIKKGLAYSTISQLGYMVMAMGIGAYSAGLFHLMTHAYFKA 354
Nqo12 T. thermophilus/1-606      241 HAATMVTAGVYLIARSSFLYSVLPDVSYAIAVVGLLTAAYGALSAFGQTDIKKIVAYSTISQLGYMFLAAGVGAYWVALFHVFTHAFFKA 330
NuoL E. coli/1-613              254 HAATMVTAGVYLIARTHGLFLMTPEVLHLVGIVGAVTLLLAGFAALVQTDIKRVLAYSTMSQIGYMFLALGVQAWDAAIFHLMTHAFFKA 343
ND5_M._musculus/1-607           248 HSSTMVVAGIFLLVRFHPLTTNNNFILTTMLCLGALTTLFTAICALTQNDIKKIIAFSTSSQLGLMMVTLGMNQPHLAFLHICTHAFFKA 337

NdhF3 T. elongatus/1-611          348 VLMMSIGSIIWNSVT---------QDLRLLGGLWSRRPISGISFLVGSAGLLAVPPL-ASFFPQAELLDTAFAQ---LPWVGGVLLLMNT 424
NdhF4 T. elongatus/1-609          348 LLFMSIGSVIMTTNS---------QDLTELGGLGERMPATSSAFVIGGLSLIGCLPL-GAFWSFYRGISYYWQT---MPWLVGLILVVNL 424
NdhF1 T. elongatus/1-656          355 MLFLGSGSVIHSMEGVVGHNPDLAQDMRYMGGLRKYMPITGATFLVGCLAISGVPPF-AGFWSKDEILGAVFHA---NPAMWLLTWLTAG 440
NdhF3 Synechocystis sp./1-615     348 ILVMCVGGIILNNVT---------QDLTQYGGLWSRRPISGLSYLVGVASLIALPPF-GTFWAWLKLAENLSAT---SPLLVGVLLVVNL 424
NdhF4 Synechocystis sp./1-634     348 LLFMSIGSIIFTTSG---------QNITEMGGLWNRMPVTTTSFVVGSAGLLAVFPL-GMFWTWQKWFSGDWLV---SWPLLALLIFVNL 424
NdhF1 Synechocystis sp./1-681     355 MLFLCSGSVIHGMEGVVGHDPILAQDMRIMGGLRKYMPITATCFLIGTLAICGIPPF-AGFWSKDEILGLAFQA---NPLLWFVGWATAG 440
Nqo12 T. thermophilus/1-606      331 LLFLASGSVIHALGG--------EQDVRKMGGLWKHLPQTRWHALIGALALGGLPLL-SGFWSKDAILAATLTYPFGGVGFYVGALLVAV 411
NuoL E. coli/1-613              344 LLFLASGSVILACHH--------EQNIFKMGGLRKSIPLVYLCFLVGGAALSALPLVTAGFFSKDEILAGAMA--NGHINLMVAGLVGAF 423
ND5_M._musculus/1-607           338 MLFMCSGSIIHSLAD--------EQDIRKMGNITKIMPFTSSCLVIGSLALTGMPFL-TGFYSKDLIIEAINTC-NTNAWALLITLIATS 417

NdhF3 T. elongatus/1-611          425 FAAFSLGRTFCLVWGGEVKPMTA---------------------------------------RSPEVFWPMILPMTVDLGLVLHLPILMA 475
NdhF4 T. elongatus/1-609          425 LTAVNLTRVFRLVFLGPAQPKTR---------------------------------------RAPEVTWPLAVPMVTLSILNMLVPFILQ 475
NdhF1 T. elongatus/1-656          441 LTAFYMFRMYFMTFEGKFRNVPPERQE-------------------------HHDHHSHHAAVPHESPWTMTLPLVVLAIPSTLIGFVGT 505
NdhF3 Synechocystis sp./1-615     425 LTAFNVTRGFCLIFGGEAKPMTV---------------------------------------RSPEGLWALVLPMVVTVGFALHLSLILK 475
NdhF4 Synechocystis sp./1-634     425 FSALNLTRVFRLVFLGKPQPKTR---------------------------------------RAPEVPWPMAVPMVSLIIVTLLVPIAPL 475
NdhF1 Synechocystis sp./1-681     441 MTAFYMFRMYFMTFEGGFRGNDQEAKDGVLQFYGLLPNFGPGAMNVKELDHEAGHDDHGHSSEPHESPLTMTFPLMALAVPSVLIGLLGR 530
Nqo12 T. thermophilus/1-606      412 LTAMYAMRWFVLVFLGEERGH-----------------------------------H-----HPHEAPPVMLWPNHLLALGSVLAGYLAL 461
NuoL E. coli/1-613              424 MTSLYTFRMIFIVFHGKEQIH-----------------------------------A-----HAVKGVTHSLPLIVLLIL-STFVGALIV 472
ND5_M._musculus/1-607           418 MTAMYSMRIIYFVTMTKPRFPPLIS--------------------------------------INENDPDLMNPIKRLAFGSIFAGFVIS 469

NdhF3 T. elongatus/1-611          476 R---------------------FDWVIWTQPSLATAALTITALLGWGVAAWVYLGKAIPKPV----QFPLPSVQN--------LLAYDFY 533
NdhF4 T. elongatus/1-609          476 R--V-----------Q---LLPETIDWTI-----VALLVVSGLAGILLGGFVTLKRSWTRPI----KVPLRFVQD--------LLAYDFY 532
NdhF1 T. elongatus/1-656          506 P--FNNLFEVFIHAPGEEKVAEHAVDLTEFL-ILGGSSVGIGLMGITVAYLMYLKGTPSPQ-AI-----AKAIQP-----LYQFSLHKWY 581
NdhF3 Synechocystis sp./1-615     476 Q--G-NLLPD-----------FADINWGL-----SSVLIASSLLGVGSSAFIYLNPKITKPI----DLPLPVVQN--------FFAYDLY 534
NdhF4 Synechocystis sp./1-634     476 Q--W-SFWLSATYPLG---LTSPVTQWA------MPLLMVAGITGILLGSLMPLRRNLSRSS----RLPVRFLQD--------LFAYDVY 541
NdhF1 Synechocystis sp./1-681     531 P--WANQFEAFIHAPGEVVEHAAEFEWGEFY-VMAGNSIGIALIGITVASLMYWQHKFDPK-VL-----AEKFPS-----LYQLSLNKWY 606
Nqo12 T. thermophilus/1-606      462 PHPLPNVLEPFLKPALAEVE-AHH------L-SLGAEWGLIALSAAVA-LLGLWAGFVFFQ--------RKVFPA-WYLAFEAASREAFY 533
NuoL E. coli/1-613              473 P-PLQGVLPQTTEL----AH-GSM------L-TLEITSGVVAVVGILL-AAWLWLGKRTLVTSIANSAPGRLLGTWWYNAW--------G 540
ND5_M._musculus/1-607           470 YNIPPTSIPVLTMP----------------W-FLKTTALIISVLGFLIALE---LNNLTMKLSMNKANPYSSFST-----------LLGF 528

NdhF3 T. elongatus/1-611          534 TPKLYRATV--------VGVVDMISRITAWFDRTFVDGTGNAFGVVTLLGGDRLKYSTTGQSQAYILTILMGIAILVI---AICWPLLA- 611
NdhF4 T. elongatus/1-609          533 IEELYRYTV--------VWAVRSLSQLSAWVDRHIVDRIVNTTGAASLVGGELLKYSASGQSQAYLLLVFIGVAILGG---AIAWLLL-- 609
NdhF1 T. elongatus/1-656          582 FDELYEAVF--------IKGCRRLARQVLEVDYNVVDGVVNLTGFVTMVTGEGLKYLQNGRAQFYALIVL-LAVLGFVI---FSVQ--T- 656
NdhF3 Synechocystis sp./1-615     535 TDKFYKLTI--------VAVIDSISRLINWFDKTFVDGVINLIGIVTIFSGQSLKYNVSGQTQFYVLSIVLGLTLIGA---FLSYSLLGQ 613
NdhF4 Synechocystis sp./1-634     542 LDKIYGATV--------VAAVAAIAKISTWFDRYVIDGIVNLVSLVTIFSGSALKYNVTGQSQFYLLTILVGVALLIWFSLSGQWMAIRQ 623
NdhF1 Synechocystis sp./1-681     607 FDDLYDKLF--------VQGSRRVARQIMEVDYKVIDGAVNLTGLVTLVSGEGLKYLENGRAQFYALIVF-GAVLGFVI---VFSL--T- 681
Nqo12 T. thermophilus/1-606      534 VDRAYNALI--------VNPLKALAEALFYGDRGLLSGYFGL-GGAARSLGQGLARLQTGYLRVYALLFVLGALLLLGV---MRW----- 606
NuoL E. coli/1-613              541 FDWLYDKVF--------VKPFLGIAWLL---KRDPLNSMMNIPAVLSRFAGKGLLLSENGYLRWYVASMSIGAVVVLAL---LMVL--R- 613
ND5_M._musculus/1-607           529 FPSIIHRITPMKSLNLSLKTSLTLLDLIW-LEKTIPKSTSTL-----HTNMTTLTTNQKGLIKLYFMSFLINIILIIIL---YSIN--LE 607

NdhF3 T. elongatus/1-611              -----------
NdhF4 T. elongatus/1-609              -----------
NdhF1 T. elongatus/1-656              -----------
NdhF3 Synechocystis sp./1-615     614 AF---------                                                                                615
NdhF4 Synechocystis sp./1-634     624 FWSSWLSLILP                                                                                634
NdhF1 Synechocystis sp./1-681         -----------
Nqo12 T. thermophilus/1-606          -----------
NuoL E. coli/1-613                   -----------
ND5_M._musculus/1-607                -----------
```

Supplementary Table 3 (*contd.*) | Multiple sequence alignment (MSA). of the CupA (top) and NdhF (bottom). Colouring conservation threshold: 50%. Colouring method: clustal. Colouring conservation threshold: 90%. The MSAs were performed with ClustalW.[8]

Supplementary Table 4 | List of primers for the construction of the *T. elongatus* CupS-TwinStrep-tag mutant.

Primer Name	DNA Sequence (5' to 3')	Description	PCR[a]
P1-*for*	AAAGAGCTCGGCCATAGCCAGATGTC	*cupS* (*tll0220*), US region and StrepII-tag incl. 5'-end SacI and 3'-end SacII restriction site, amplified from genomic DNA	1,975
P2-*rev*	GGGCCGCGGTTATTTTTCGAACTGCGGGTGGCTCCAAGCGCTGACTTTGTAGGGAATGTTG		
P3-*for*	GGGCTGCAGGGTATTAGCAGATTTCCC	*cupS (tll0220)* and DS region incl. 5'-end PstI and 3'-end KpnI restriction site, amplified from genomic DNA	1,512
P4-r*ev*	CCCGGTACCCAATCAGGTCGTAGTTGC		
P5-*rev*	GCAGAACCACCAGAACCACCGCCGCTGCCGCCGCCTTTTCGAACTGCGGGTGGC	*cupS* (*tll0220*), US region and 1/2 TS-tag incl. 5'-end SacI and 3'-end SacII restriction site, amplified from pCupS-StrepII-tag (with P1-*for*)	1,998
P6-*rev*	CCCGCGGCCGCTTACTTCTCAAATTGCGGATGAGACCACGCAGAACCACCAGAACCACC	*cupS* (*tll0220*), US region and TS-tag incl. 3'-end NotI restriction site, amplified from product P1/P5 (with P1-*for*)	2,037
P7-*for*	GGATTAACGGACGCGATTAC	Segregation check	2,148 (WT 842)
P8-*rev*	AGGCCGATAAGGCAGAACTA		

[a] PCR product length in basepair, US = Upstream, DS = Downstream

Supplementary References

1. Iwai M., Katoh H., Katayama M. & Ikeuchi M. Improved genetic transformation of the thermophilic cyanobacterium, *Thermosynechococcus elongatus* BP-1. *Plant Cell Physiol.* **45**, 171–175 (2004).
2. Yamaoka, T. Satoh, K. & Katoh S. Photosynthetic activities of a thermophilic blue-green alga. *Plant Cell Physiol.*, **19**, 943–954 (1978).
3. Rippka, R., Deruelles, J., Waterbury, J. B., Herdman, M. & Stanier, R. Y. Generic Assignments, Strain Histories and Properties of Pure Cultures of Cyanobacteria. *Microbiology* **111**, 1–61 (1979).
4. Schuller, J. M. *et al.* Structural adaptations of photosynthetic complex I enable ferredoxin-dependent electron transfer. *Science*, **363**, 257–260 (2019).
5. Wulfhorst, H., Franken, L. E., Wessinghage, T., Boekema, E. J. & Nowaczyk, M. M. The 5 kDa protein NdhP is essential for stable NDH-1L assembly in Thermosynechococcus elongatus. *PLoS One* **9**, e103584 (2014).
6. Kuhl, H. *et al.* Towards Structural Determination of the Water-splitting Enzyme. *J. Biol. Chem.* **275**, 20652–20659 (2000).
7. Sinclair, S.A., Larue, C., Bonk, L., Khan, A., Castillo-Michel, H., Stein, R.J., Grolimund, D., Begerow, D., Neumann, U., Haydon. M.J., Krämer. U. Etiolated Seedling Development Requires Repression of Photomorphogenesis by a Small Cell-Wall-Derived Dark Signal Curr Biol. 27, 3403-3418 (2017).
8. Sievers, F. *et al.* Fast, scalable generation of high-quality protein multiple sequence alignments using Clustal Omega. *Mol. Syst. Biol.* **7,** 1-6 (2011).
9. Korste, A., Wulfhorst, H., Ikegami, T., Nowaczyk, M.M. & Stoll, R. Solution structure of the NDH-1 complex subunit CupS from Thermosynechococcus elongatus. *Biochim. Biophys. Acta - Bioenerg.* **1847**, 1212–1219 (2015).
10. Agip, A. N. A. *et al.* Cryo-EM structures of complex I from mouse heart mitochondria in two biochemically defined states. *Nat. Struct. Mol. Biol.* **25**, 1–9 (2018).
