# Supplementary material for: Redox-coupled proton pumping drives carbon concentration in the photosynthetic complex I
Source: Nat Commun. 2020 Jan 24;11:494. doi: 10.1038/s41467-020-14347-4 (PMC6981117; doi:10.1038/s41467-020-14347-4)
Supplement: Supplementary file 3 — Description of Additional Supplementary Files [file 41467_2020_14347_MOESM3_ESM.pdf]

Title: Supplementary Movie 1
Description: Architecture of the CO_2-concentrating photosynthetic complex I
